# Supplementary material for: Myristoyl-CM4 Exhibits Direct Anticancer Activity and Immune Modulation in Hepatocellular Carcinoma: Evidence from In Vitro and Mouse Model Studies
Source: Int J Mol Sci. 2025 Apr 18;26(8):3829. doi: 10.3390/ijms26083829 (PMC12028079; doi:10.3390/ijms26083829)
Supplement: Supplementary file 1 [file ijms-26-03829-s001.zip › FIG S1.pdf]

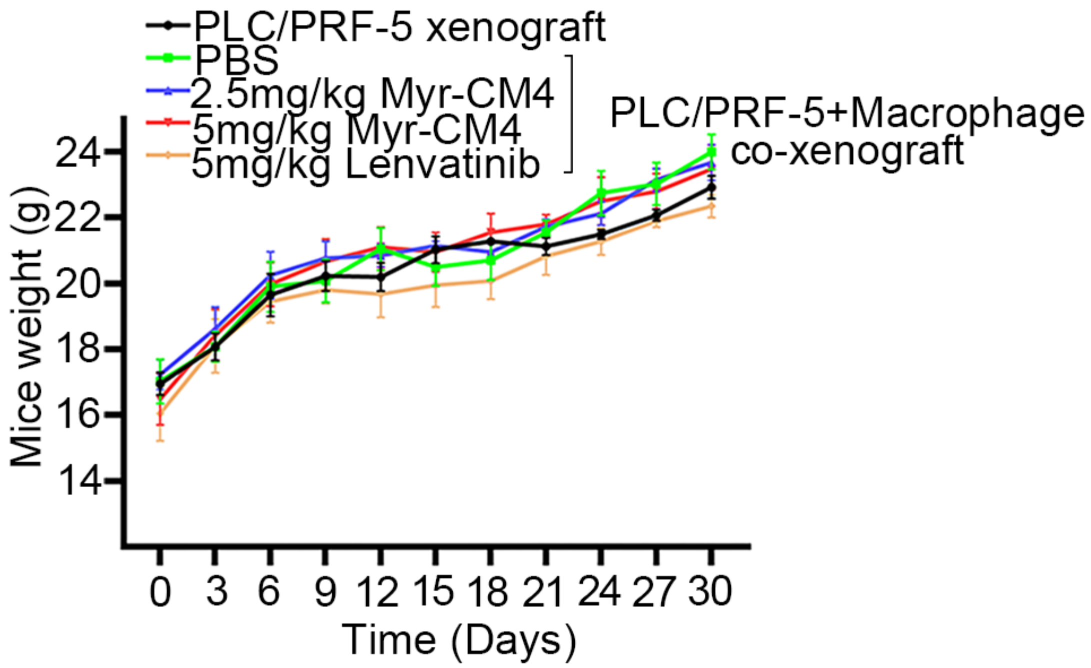

Figure S1. PLC/PRF-5 cells and M0 macrophages were injected subcutaneously into nude mice (4-6 weeks) to form a co-xenograft mouse model and divided into four groups: PBS group, 2.5 mg myristoyl-CM4 group, 5 mg myristoyl-CM4 group, and 5 mg Lenvatinib group. Body weight was determined every 3 days and tumor growth curves over time was illustrated.
