# Supplementary material for: Myristoyl-CM4 Exhibits Direct Anticancer Activity and Immune Modulation in Hepatocellular Carcinoma: Evidence from In Vitro and Mouse Model Studies
Source: Int J Mol Sci. 2025 Apr 18;26(8):3829. doi: 10.3390/ijms26083829 (PMC12028079; doi:10.3390/ijms26083829)
Supplement: Supplementary file 1 [file ijms-26-03829-s001.zip › Table S1.pdf]

Table S1

	Gene	Sequence（5′-3′）
qRT-PCR	iNOS	F: CATCCTCTTTGCGACAGAGAC
		R: GCAGCTCAGCCTGTACTTATC
	CD86	F: CCATCAGCTTGTCTGTTTCATTCC
		R: GCTGTAATCCAAGGAATGTGGTC
	CD163	F: GAGGAGGCAGAAGAATGGT
		R: CCACTTCACAGGTGAGGGAC
